# Gradual transition from mosaic to global DNA methylation patterns during deuterostome evolution

**DOI:** 10.1186/1471-2105-11-S7-S2

**Published:** 2010-10-15

**Authors:** Kohji Okamura, Kazuaki A Matsumoto, Kenta Nakai

**Affiliations:** 1 Human Genome Centre, Institute of Medical Science, University of Tokyo, 4-6-1 Shirokanedai, Minato Ward, Tokyo 108-8639, Japan; 2 Centre for Informational Biology, Ochanomizu University, 2-1-1 Otsuka, Bunkyo Ward, Tokyo 112-8610, Japan; 3 Department of Electric Engineering and Bioscience, Graduate School of Sciences and Engineering, Waseda University, 2-2 Wakamatsu-cho, Shinjuku Ward, Tokyo 162-8480, Japan; 4 Institute for Bioinformatics Research and Development, Japan Science and Technology Agency, 4-1-8 Honcho, Kawaguchi 332-0012, Japan

## Abstract

**Background:**

DNA methylation by the Dnmt family occurs in vertebrates and invertebrates, including ascidians, and is thought to play important roles in gene regulation and genome stability, especially in vertebrates. However, the global methylation patterns of vertebrates and invertebrates are distinctive. Whereas almost all CpG sites are methylated in vertebrates, with the exception of those in CpG islands, the ascidian genome contains approximately equal amounts of methylated and unmethylated regions. Curiously, methylation status can be reliably estimated from the local frequency of CpG dinucleotides in the ascidian genome. Methylated and unmethylated regions tend to have few and many CpG sites, respectively, consistent with our knowledge of the methylation status of CpG islands and other regions in mammals. However, DNA methylation patterns and levels in vertebrates and invertebrates have not been analyzed in the same way.

**Results:**

Using a new computational methodology based on the decomposition of the bimodal distributions of methylated and unmethylated regions, we estimated the extent of the global methylation patterns in a wide range of animals. We then examined the epigenetic changes *in silico *along the phylogenetic tree. We observed a gradual transition from fractional to global patterns of methylation in deuterostomes, rather than a clear demarcation between vertebrates and invertebrates. When we applied this methodology to six piscine genomes, some of which showed features similar to those of invertebrates.

**Conclusions:**

The mammalian global DNA methylation pattern was probably not acquired at an early stage of vertebrate evolution, but gradually expanded from that of a more ancient organism.

## Background

In mammals, DNA methylation is achieved by the collaboration of several DNA methyltransferases; *i.e.*, Dnmt1 and the Dnmt3 family [1]. It plays important roles in general gene silencing, the repression of one of the two alleles of imprinted genes and X-linked genes in females, and the inactivation of various transposons. Whereas the Dnmt family is not found in some model organisms, such as the budding yeast, nematode, and fruit fly, in which DNA methylation is absent or below the limit of detection, it is well conserved among deuterostomes [2]. However, functional analyses of the methylation have been limited to mammals; *i.e.*, mouse and human. In contrast, the DNA methylation status has been investigated widely, from mammals to invertebrate deuterostomes, such as the sea urchin, revealing that large parts of these genomes are subject to stable methylation, even in invertebrates [3]. Methylated and unmethylated DNA sequences coexist and pattern the genomes of these animals. Unmethylated stretches in vertebrates are mainly limited to CpG islands, which have been used as gene markers [4].

It is well documented that the global patterns and levels of DNA methylation are distinct between vertebrates and invertebrate deuterostomes [5]. In the former group, almost every CpG site is methylated, with the exception of those in CpG islands. For instance, nearly 80% of CpG sites are methylated in the human genome [6]. Because CpG sites are excessively concentrated in CpG islands, we may infer that almost all other parts are methylated regions. This is called global pattern of DNA methylation. In contrast, the genome of the sea squirt, an ascidian, for instance, contains roughly equal amounts of methylated and unmethylated regions [7]. Relatively long genomic tracts of tens or hundreds of kilobases are hypermethylated, and other long tracts are hypomethylated. The alternation of these two distinctive types of tracts is called fractional or mosaic pattern of DNA methylation. DNA methylation analyses of the sea urchin, lancelet, ascidian, lamprey, and hagfish have suggested that the transition from the mosaic to the global methylation pattern occurred at an early stage of vertebrate evolution [5,8].

Curiously, especially to computational biologists, methylated and unmethylated regions can be reliably predicted from the local frequency of CpG dinucleotides [9]. Separated by relatively sharp boundaries, methylated and unmethylated regions tend to contain few and many CpG sites, respectively. This may be attributable to the inherent mutability of 5-methylcytosine [10] and seems to be consistent with our knowledge of the methylation status of CpG islands and other regions in the mammalian genomes [11].

So far, however, DNA methylation patterns and methylation levels have not been analyzed in the same way in vertebrates and invertebrates. The prevalent view that the DNA methylation of the two groups differs seems to have hindered us from examining their common features, despite the use of orthologous enzymes in both systems. It is unlikely that the change in the methylation pattern happened suddenly at a specific evolutionary stage. An abrupt change in the pattern of methylation could be deleterious to organisms. If a gradual transition from the mosaic to the global methylation pattern occurred, traces of the transition might be apparent in extant organisms located evolutionarily near the transition zone; *e.g*., fishes and invertebrate chordates.

To test this possibility of a gradual transition, we developed a new computational methodology to estimate the distributions of methylated and unmethylated regions in various animal genomes. This methodology does not confidently predict the methylation status or level in a specific region, but predicts the proportion of methylated and unmethylated regions from local frequencies of CpG dinucleotides. It is well known that in genomic imprinting and X-inactivation, one member of a pair of alleles with identical sequences sometimes exhibits a different level of DNA methylation, and these variations in methylation levels are often dependent on the developmental stages, tissues, or cell types [12]. The levels also vary among various cancer cells [13]. Heavily methylated CpG islands [14] and long unmethylated tracts with low CpG contents [15] have also been documented. Recent research has suggested that specific sequence motifs are more important than CpG contents in the establishment of proper DNA methylation [16]. These facts make us reluctant to predict methylation levels. However, because these observations are exceptional and infrequent compared with the general features observed in whole genomes, the proportion of DNA methylation may be stochastically estimated in each species. Experimental data for the human and ascidian genomes demonstrated the plausibility of this methodology. We applied the method to the analysis of deuterostomes, from the sea urchin through to the human to evaluate the extent of the global methylation pattern, and investigated the global changes in DNA methylation pattern *in silico*.

## Results

**Figure 1:**
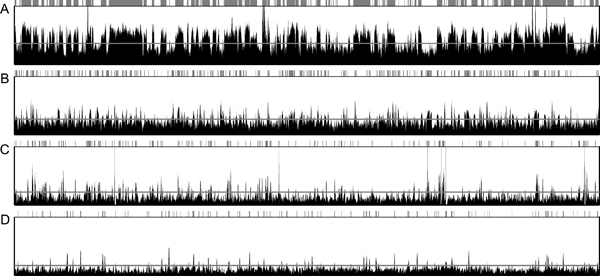
**CpG-score changes along the genomic coordinates**. Genomic regions of 2-Mb were arbitrarily selected from the (A) ascidian (sea squirt) chr02q 3,857,310-5,857,309, (B) zebrafish chr1 21,198,157-23,198,156, (C) frog scaffold_1 4,936,533-6,936,532, and (D) human chr18 42,391,708-44,391,707. The horizontal lines crossing the graph are the barycenters of the two normal distributions (see Figure 3). Unmethylated regions are indicated by shaded tracts above the graph. Typical mosaic and global patterns are seen in the ascidian and human genomes, respectively.

To identify typical patterns of mosaic and global DNA methylation, we first arbitrarily chose four 2-Mb contiguous regions from four distinctive species and drew the changes in the ratios of observed over expected CpG numbers, hereafter called the "CpG score" (as in the UCSC database), along the genomic coordinates (Figure 1). A mosaic pattern is seen in the ascidian genome, characterized by the frequent appearance of broad crests of presumably unmethylated regions. In contrast, a global pattern is apparent in the human genome, characterized by sharp sparsely distributed peaks. The peaks presumably correspond to CpG islands, which are generally unmethylated. The patterns seen in the zebrafish and frog genomes appear intermediate between those of the ascidian and human.

**Figure 2:**
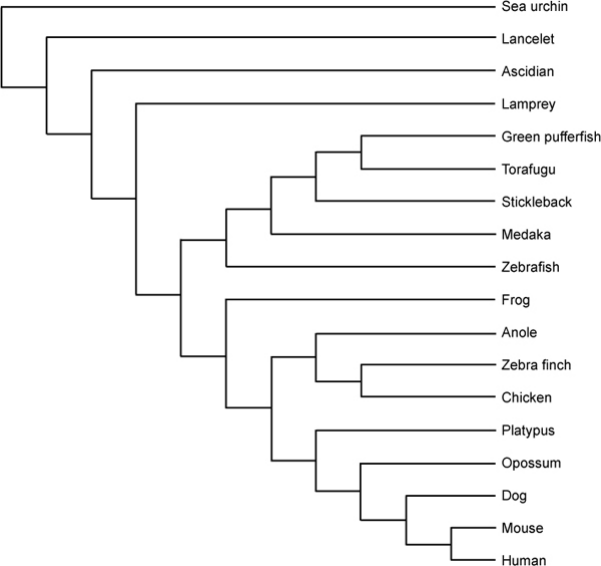
**Schematic representation of the phylogenetic relationships among deuterostomes**. In total, 26 deuterostome genomes were examined in the present study. Out of them, 18 species are schematically represented here [26,31]. Human is placed at the bottom. The upper species are placed, the further they are diverged from human. As for teleosts, they are ordered in accordance with the two ratio (Tables 1 and 2).

**Figure 3:**
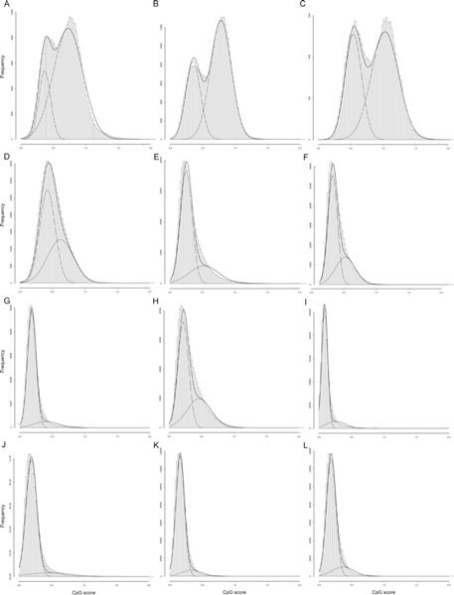
**Histograms showing the CpG-score frequencies in 2-kb genomic fragments**. Each histogram covers the whole genome of the (A) sea urchin, (B) lancelet (amphioxus), (C) ascidian, (D) zebrafish, (E) frog, (F) anole (a kind of lizard), (G) chicken, (H) platypus, (I) opossum, (J) dog, (K) mouse, or (L) human. Apparent bimodal distributions are seen in the invertebrate deuterostomes. All distributions were compulsorily separated into two normal distributions. The two decomposed Gaussian curves and a merged curve are also drawn on each histogram.

Among the deuterostomes, we chose the genomes of *Strongylocentrotus purpuratus *(purple sea urchin), *Branchiostoma floridae *(Florida lancelet), *Ciona intestinalis *(ascidian), *Danio rerio *(zebrafish), *Xenopus tropicalis *(western clawed frog), *Anolis carolinensis *(green anole), *Gallus gallus *(chicken), *Ornithorhynchus anatinus *(platypus), *Monodelphis domestica *(gray short-tailed opossum), *Canis familiaris *(dog), *Mus musculus *(mouse), and *Homo sapiens *(human) to represent various clades, namely, echinoderms, cephalochordates, urochordates, fishes, amphibians, reptiles, avians, monotremes, marsupials, carnivores, rodents, and primates (Figure 2). We then examined the genome-wide distributions of the CpG scores for each of them, with a sliding window of 2 kb. In animals with a mosaic pattern, a bimodal distribution can be seen, arising from comparable amounts of low- and high-CpG-score regions. In other animals, only the low-CpG band is conspicuous, reflecting a global methylation pattern (Figure 3).

A similar analysis of CpG-score distributions has been performed for 3 invertebrate and 6 vertebrate genomes [17]. Those authors showed markedly different distributions of CpG scores in the promoter and intronic sequences. Our results for whole genomes are congruent with their distributions in intronic sequences. It has been reported that invertebrate and vertebrate genomes show bimodal and unimodal distributions, respectively. Using the NOCOM software with an expectation maximization algorithm to fit the distribution data [18], Elango and Yi revealed that, in invertebrates, methylated and unmethylated regions show discrete normal distributions with low and high CpG scores, respectively. Although the vertebrate distributions were assumed to be unimodal, we noted that the bell shapes were not symmetrical. The right sides of the curves, which correspond to higher CpG scores, bulged slightly. This could be caused by the distribution of CpG scores in unmethylated regions. Indeed, CpG islands, which are considered unmethylated regions in general, cover only 0.69% and 0.39% of the human and mouse genomes, respectively (see Methods). When we applied the NOCOM software to each seemingly unimodal distribution to separate it compulsory into two distinct normal distributions, better fits were obtained (Figure 3).

**Figure 4:**
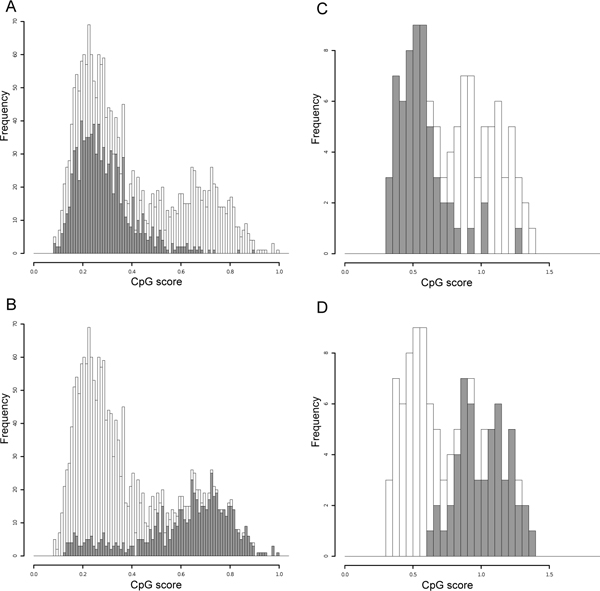
**Histograms showing the CpG-score frequencies in 2-kb genomic fragments obtained from experimental data for the DNA methylation levels of the human (A and B) and ascidian (C and D) genomes**. The distributions of hypermethylated (A and C) and hypomethylated (B and D) fragments (gray) are shown with the whole data (white). In the human data, the right peaks are increased because of the preferential selection of CpG islands in the Human Epigenome Project.

It is likely that these two components separately represent the CpG-score distributions of putative methylated and unmethylated regions. To demonstrate the plausibility of dividing the single distribution into two normal distributions, we examined the distributions of experimentally verified hypermethylated and hypomethylated regions. We first used methylation data provided by the Human Epigenome Project [19,20]. For each 2-kb window, the CpG score and the average of methylation level were calculated and represented as histograms. We assumed that windows with averages of 70%-100% and 0%-30% are hypermethylated and hypomethylated regions, respectively (Figure 4). Both of the regions had bell-shaped distributions, supporting the plausibility of this method. Compared with Figure 3, the right peak is somewhat protruding because of the preferential selection of CpG islands in the Human Epigenome Project. We also drew similar histograms for two 1-Mb genomic regions of the ascidian genome analyzed by methylation-sensitive PCR [9]. Although the number of data was not large, the histograms look bell-shaped (Figure 4). The implication of these results is that the CpG score cannot predict the methylation level in a specific region, but can stochastically identify the ratio of methylated to unmethylated regions. For instance, there are some hypomethylated regions with low CpG scores and the extent of these regions can be estimated by fitting them to a normal distribution.

We thought that the decomposition method should allow us to draw a line between the two regions in each organism. In ascidians, for example, a CpG score of 0.8 was used as the demarcation to distinguish methylated and unmethylated regions [9]. In addition to the uncertainty implicit in using this fixed value, another concern is whether or not a fixed value can be applied to all organisms with different G+C and CpG contents. We conceived an impartial way in which the barycenter of the two normal distributions could be used as the decision boundary for each species. In this way, we can separate the methylated and unmethylated regions with confidence species by species. The barycenter calculated for the whole ascidian genome is 0.721, which shows satisfactory agreement with the value used in the preceding study. The barycenters for all the species are tabulated (Table 1) and four of them are drawn on the line graphs of the CpG scores (Figure 1).

**Table 1:** Statistical data on the methylated and unmethylated regions in deuterostomes

		Methylated regions		Unmethylated regions			
Deuterostome species	Boundary (CpG score)	Mean (bp)	SD (bp)	Mean (bp)	SD (bp)	Ratio of lengths	Ratio of SDs
Sea urchin	0.436	3635.6	3944.7	6554.9	11928.6	1.803	3.024
Lancelet	0.489	4761.9	4982.9	7689.1	11142.2	1.615	2.236
Ascidian	0.721	5314.6	5570.8	6785.9	9550.2	1.277	1.714
Zebrafish	0.535	5401.0	5695.4	3402.7	3206.9	0.630	0.563
Frog	0.448	7678.6	8523.9	2190.2	1608.9	0.285	0.189
Anole	0.332	10050.1	11854.7	3275.2	3650.7	0.326	0.308
Chicken	0.375	20112.8	27862.0	2359.5	1931.9	0.117	0.069
Platypus	0.368	8584.3	12368.2	3958.6	5626.0	0.461	0.455
Opossum	0.233	26111.6	35360.4	2640.6	2626.1	0.101	0.074
Dog	0.432	39580.4	71275.6	3105.2	3588.5	0.078	0.050
Mouse	0.318	38228.3	57618.0	2387.5	1657.3	0.062	0.029
Human	0.340	25535.8	38772.9	2621.2	2715.3	0.103	0.070

**Figure 5:**
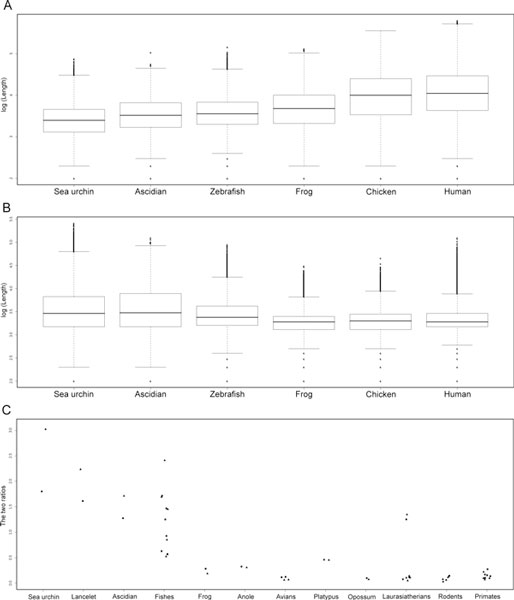
**Lengths of methylated (A) and unmethylated (B) regions and the two ratios (C) that can be used as indices of the global methylation pattern**. The lengths are shown on a log scale. The consistent tendencies in the lengths and scatter of the lengths can be seen to parallel the course of deuterostome evolution. The ratios of the lengths are indicated by squares and the ratios of the standard deviations of the lengths are by triangles. Laurasiatherians include cow, horse, dog, and cat. Exceptionally high ratios were observed in the cat genome.

Using the method described in the Methods section, we then calculated the lengths of methylated and unmethylated regions for all the species. Because the scatter of the length values is also an indicator of the extent of the mosaic pattern, we also calculated the standard deviations of the lengths (Table 1). As expected, there are consistent tendencies observed along the phylogenetic tree of the deuterostomes [21]. The more primitive the organism, the shorter are the methylated regions and the longer are the unmethylated regions. The more primitive the organisms, the smaller are the standard deviations of the lengths of methylated regions and the larger are the standard deviations of the lengths of the unmethylated regions (Figure 5). To introduce more readable indices, we divided the lengths or standard deviations of the unmethylated regions by those of the methylated regions. The more primitive the organisms, the larger are both of these ratios (Table 1). The two ratios are approximately 1.0 for the ascidian genome, in which the amounts of methylated and unmethylated are similar. The DNA methylation pattern of the sea urchin genome has a length ratio of 1.803 and a standard deviation ratio of 3.024, so it might be patchy rather than mosaic.

The boundary, the ratio of lengths, and the ratio of standard deviations can be used to compare global DNA methylation patterns numerically and objectively. We developed software to calculate these three indices for any genomic sequences. Both CGI and stand-alone versions are available at http://epigenetics.hgc.jp/mosaicglobal/. Using this software, we calculated the three indices for *Taeniopygia guttata *(zebra finch) to be 0.299, 0.124, and 0.063, respectively, which are very similar to those of the chicken (Figure 5). We also obtained the indices for five additional fishes: *Petromyzon marinus *(sea lamprey), *Oryzias latipes *(Japanese medaka), *Gasterosteus aculeatus *(three-spined stickleback), *Takifugu rubripes *(torafugu), and *Tetraodon nigroviridis *(spotted green pufferfish). Intriguingly, the two ratios show a wide range of values in these non-tetrapod vertebrates (Table 2). Finally, we obtained the indices for other mammalian genomes. At this point, we have analyzed all deuterostome genomes available at http://genome.ucsc.edu/ (Figure 5).

**Table 2:** Three indices of fishes used to compare their global methylation patterns

Fish species	Boundary (CpG score)	Ratio of lengths	Ratio of SDs
Lamprey	0.768	0.928	0.852
Green pufferfish	0.533	1.717	2.412
Torafugu	0.486	1.469	1.693
Stickleback	0.589	1.252	1.453
Medaka	0.498	0.573	0.524
Zebrafish	0.535	0.630	0.563

## Discussion

A bimodal distribution of CpG scores has been reported for mouse and human promoter sequences [11,22,23]. However, when we turn our attention to their distribution in the whole genome, the distribution of unmethylated regions, *i.e.*, CpG islands, is negligible and is clearly covered by the distribution of the methylated regions (Figure 3). In the present study, we have demonstrated that the distributions of the methylated and unmethylated regions in whole genomes can be represented by the composition of the two discrete normal distributions. This is supported by experimental data (Figure 4) and overlaying the two distributions produces a better fit. It is not easy to predict DNA methylation levels from local genomic sequences only. Moreover, the status can differ depending on developmental stage, tissue, and cell type. Nevertheless, the implication of the present findings is that the overall patterns formed by sets of each methylation status can be stochastically estimated by fitting them to two normal distributions. This computational methodology allowed us to observe the transition to the global DNA methylation pattern during deuterostome evolution. Evolutionary changes in 5-methylcytocine levels have been investigated in detail and in a large number of species [24]. However, what we have observed here are not average levels but the transition of patterns that are formed by methylated and unmethylated regions.

It has been widely believed that cephalochordates, including lancelets, are more closely related to vertebrates than to urochordates, represented by ascidians [25]. This view may have caused researchers to overlook the gradual transition in the DNA methylation pattern, even though they observed higher levels of methylation in the ascidian than in the lancelet [5]. Recent studies of chordate genomes have changed the conventional consensus [26,27]. Currently, both urochordates and vertebrates are thought to have evolved from a common ancestor of cephalochordate-like organisms. Consistent with this, our results indicate a firm direction in the changes in the methylation pattern during evolution, based on the assumption that lower organisms have relatively better retained their primitive features than higher ones.

To take a closer look at this transition, we focused on six fish species. The existence of CpG islands in fish genomes is unclear, but an analysis of evidence-based transcription start sites has clearly shown that, like mammals, there are a large number of promoter-associated CpG islands in the medaka genome [28]. However, because sequence features, including the G+C and CpG contents, seem to vary considerably among fishes, the definition of fish CpG islands is still controversial [29]. Adjustable criteria that do not rely on *ad hoc *thresholds may be required to define standardized CpG islands among highly divergent species. In the present study, CpG islands were not considered because we wanted to give organisms lacking CpG islands equal attention. Instead, the boundaries between methylated and unmethylated regions were defined by analyzing the CpG-score distributions, species by species. Although the indices obtained showed a wide range, they are consistent with the piscine phylogeny (Figure 2) [30], and are situated between those of invertebrates and tetrapods, supporting a gradual transition in the methylation pattern (Table 2). The two pufferfish genomes show exceptionally high ratios. This could be attributable to their excessively reduced genomes relative to those of other vertebrates [31]. A similar tendency was also observed in platypus, which is a mammal that exhibits some characteristics of reptiles, probably because of the unique character of its genomic sequence [32]. Exceptionally high ratios observed in cat could be ascribed to its low coverage of 1.9 fold (Figure 5) [33]. Its finished sequences are eagerly awaited to know the *bona fide *causes. We intend to examine the causes of these discrepancies. The genomes of increasing numbers of organisms have been sequenced [34]. The boundary and the indices used to estimate the extent of the global methylation pattern will be fundamental values in future comparative studies.

## Conclusions

In mammals, DNA methylation is essential for normal development [12,16,35]. A wave of *de novo *methylation occurs globally around the time of implantation. Interestingly, we observed a transition to the global DNA methylation pattern from the invertebrates to mammals. These facts recall Haeckel's dictum "Ontogeny recapitulates phylogeny." From our computational analysis, it appears that the establishment of global DNA methylation during ontogeny recapitulates phylogeny. Although the methodology is in part supported by experimental data, our conclusion must be confirmed experimentally. The distributions of CpG scores, which are the fundamental data in the present study, might be the consequences of the spontaneous deamination of methylated CpG sites, rather than the cause of the established methylation levels. The methylation and unmethylation signals of various deuterostomes are yet to be identified in DNA methylation analyses. They are eagerly awaited. However, by using a new computational methodology, our study has shed light on the unexplored molecular evolution of epigenetics.

## Methods

### Genomic sequences of deuterostomes

We downloaded the genomic sequence data from the UCSC ftp site ftp://hgdownload.cse.ucsc.edu/. The names of the assemblies used in the analysis are strPur2 for *Strongylocentrotus purpuratus*, braFlo1 for *Branchiostoma floridae*, ci2 for *Ciona intestinalis*, petMar1 for *Petromyzon marinus*, tetNig1 for *Tetraodon nigroviridis*, fr2 for *Takifugu rubripes*, gasAcu1 for *Gasterosteus aculeatus*, oryLat2 for *Oryzias latipes*, danRer4 for *Danio rerio*, xenTro2 for *Xenopus tropicalis*, anoCar1 for *Anolis carolinensis*, taeGut1 for *Taeniopygia guttata*, galGal3 for *Gallus gallus*, ornAna1 for *Ornithorhynchus anatinus*, monDom4 for *Monodelphis domestica*, bosTau4 for *Bos Taurus*, equCab2 for *Equus caballus*, canFam2 for *Canis familiaris*, felCat3 for *Felis catus*, cavPor3 for *Cavia porcellus*, rn4 for *Rattus norvegicus*, mm8 for *Mus musculus*, calJac1 for *Callithrix jacchus*, rheMac2 for *Macaca mulatta*, ponAbe2 for *Pongo pygmaeus*, panTro2 for *Pan troglodytes*, and hg18 for *Homo sapiens*. The ratio of the observed number to the expected number of CpG, called the "CpG score" in this paper, was calculated using 2-kb fixed lengths, with no space between two adjacent windows [36]. If a window contained more than 50 undetermined or ambiguous nucleotides, it was discarded. A large number of 2-kb sequences spanning gaps were also discarded under this criterion. Most of the analyses in this study were performed using Perl scripts, which are available upon request.

### Compulsory decomposition of the bimodal CpG-score distributions

The compulsory decomposition of the bimodal CpG-score distributions was performed with the NOCOM software [37]. Our software, designed to calculate the extent of the global methylation pattern, also includes the software that was coded in FORTRAN 77. We modified its interface so that it can be called from our Perl scripts of both stand-alone and CGI versions.

### Occupancy ratios of CpG islands in the whole genomes

We downloaded the CpG island annotation data from ftp://hgdownload.cse.ucsc.edu/apache/htdocs/goldenPath/hg18/database/cpgIslandExt.txt.gz and ftp://hgdownload.cse.ucsc.edu/apache/htdocs/goldenPath/mm8/database/cpgIslandExt.txt.gz and summed all the lengths. We then divided these by the whole lengths of hg18 (3,107,677,273 bp) and mm8 (2,664,455,088 bp), respectively.

### DNA methylation data for the human and ascidian genomes

We used the DNA methylation data of Human Epigenome Project Release 26th June 2006 [19,20]. We used sliding 2-kb windows without any overlap, to avoid including gapped regions. We considered windows with average DNA methylation levels 70%-100% and 0%-30% as hypermethylated and hypomethylated regions, respectively. For the ascidian genome, we used data obtained with methylation-sensitive PCR for two 1-Mb genomic regions [9]. The genomic coordinates of the 2-kb windows and their methylation status are tabulated (Additional file 1: Table S1).

### Estimation of the extent of the global DNA methylation pattern

We used the barycenter of the two decomposed normal distributions as the boundary of the methylated and unmethylated regions. In this way, every sliding window of 2 kb, moved in steps of 100 bp, was assigned to one of the two regions, producing binary data for each 100 bp covering the whole genomes. To smooth the binary data, a 2-kb sliding window analysis was performed again and the methylation status was decided based on whether methylation was dominant over unmethylation or *vice versa*. Consecutive statuses were clustered into a methylated or unmethylated region and the whole set of the lengths was used for the final statistical analysis to estimate the extent of global methylation pattern.

## Competing interests

The authors declare that they have no competing interests.

## Authors' contributions

KO conceived of the study, designed the study, and drafted the manuscript. KAM designed the study and analyzed the data. KN participated in the coordination of the study and helped to draft the manuscript. All authors read and approved the final manuscript.

## Supplementary Material

Additional file 1 **Table S1. The genomic coordinates and their methylation status for the ascidian DNA methylation analysis**. The ci2 chromosome name, start position, end position, methylation status, and CpG score are tabulated. Methylated and unmethylated statuses are represented by 1 and 0, respectively. Click here for file
