# Supplementary material for: Gradual transition from mosaic to global DNA methylation patterns during deuterostome evolution
Source: BMC Bioinformatics. 2010 Oct 15;11(Suppl 7):S2. doi: 10.1186/1471-2105-11-S7-S2 (PMC2957685; doi:10.1186/1471-2105-11-S7-S2)
Supplement: Additional file 1 — Table S1. The genomic coordinates and their methylation status for the ascidian DNA methylation analysis. The ci2 chromosome name, start position, end position, methylation status, and CpG score are tabulated. Methylated and unmethylated statuses are represented by 1 and 0, respectively. [file 1471-2105-11-S7-S2-S1.pdf]

Table S1. The genomic coordinates and their methylation status by the ascidian DNA methylation analysis

UCSC ci2	Start	End	Methylated	CpG score
chr04q	3184251	3186250	0	0.937
chr04q	3205251	3207250	0	0.925
chr04q	3222501	3224500	1	0.507
chr04q	3243251	3245250	0	0.900
chr04q	3249001	3251000	0	1.063
chr04q	3258751	3260750	0	0.816
chr04q	3305001	3307000	0	1.238
chr04q	3349501	3351500	0	1.253
chr04q	3357751	3359750	1	0.469
chr04q	3375751	3377750	0	NA
chr04q	3385501	3387500	1	0.903
chr04q	3389751	3391750	0	1.289
chr04q	3402001	3404000	1	NA
chr04q	3412751	3414750	0	1.011
chr04q	3419001	3421000	1	0.406
chr04q	3454251	3456250	1	NA
chr04q	3478001	3480000	1	0.424
chr04q	3494251	3496250	0	0.882
chr04q	3513001	3515000	0	1.019
chr04q	3523251	3525250	1	0.515
chr04q	3533751	3535750	0	0.849
chr04q	3540751	3542750	1	0.748
chr04q	3547501	3549500	0	1.131
chr04q	3560251	3562250	1	0.404
chr04q	3573001	3575000	0	0.864
chr04q	3581751	3583750	1	0.671
chr04q	3586001	3588000	1	0.685
chr04q	3596001	3598000	0	1.157
chr04q	3612251	3614250	0	0.887

chr04q	3619251	3621250	0	NA
chr04q	3624251	3626250	0	1.071
chr04q	3634001	3636000	1	0.388
chr04q	3640251	3642250	0	0.683
chr04q	3644001	3646000	0	1.317
chr04q	3654251	3656250	1	0.572
chr04q	3662001	3664000	0	1.108
chr04q	3671001	3673000	1	0.383
chr04q	3679501	3681500	1	0.554
chr04q	3690001	3692000	0	0.977
chr04q	3714001	3716000	0	1.142
chr04q	3740501	3742500	0	0.901
chr04q	3745501	3747500	1	0.344
chr04q	3754501	3756500	1	0.534
chr04q	3760751	3762750	0	NA
chr04q	3769001	3771000	1	0.442
chr04q	3774251	3776250	0	1.131
chr04q	3789251	3791250	1	0.624
chr04q	3857251	3859250	0	0.980
chr04q	3859751	3861750	1	0.632
chr04q	3887751	3889750	0	1.216
chr04q	3917251	3919250	1	0.512
chr04q	3945751	3947750	1	0.371
chr04q	3951501	3953500	0	1.191
chr04q	3962001	3964000	1	0.432
chr04q	3968001	3970000	0	NA
chr04q	3971251	3973250	1	0.636
chr04q	3979501	3981500	0	0.949
chr04q	3990001	3992000	1	0.471
chr04q	3996001	3998000	0	1.225
chr04q	4003001	4005000	1	0.423
chr04q	4008751	4010750	0	0.838
chr04q	4019001	4021000	1	0.498

chr04q	4043251	4045250	0	NA
chr04q	4047001	4049000	1	NA
chr04q	4062251	4064250	0	1.066
chr04q	4072751	4074750	0	0.824
chr04q	4098751	4100750	1	0.362
chr04q	4113501	4115500	0	1.089
chr04q	4120251	4122250	1	0.318
chr04q	4140251	4142250	0	0.604
chr07q	8001	10000	1	0.533
chr07q	17251	19250	1	NA
chr07q	26001	28000	1	0.570
chr07q	38251	40250	1	1.285
chr07q	68501	70500	0	NA
chr07q	101251	103250	1	1.019
chr07q	134251	136250	0	NA
chr07q	142251	144250	1	0.580
chr07q	153251	155250	1	0.579
chr07q	164251	166250	1	0.399
chr07q	168251	170250	0	1.142
chr07q	176251	178250	1	0.481
chr07q	182501	184500	0	1.384
chr07q	188251	190250	1	0.529
chr07q	208501	210500	1	0.453
chr07q	239001	241000	0	0.671
chr07q	240251	242250	1	0.376
chr07q	249501	251500	1	1.007
chr07q	281501	283500	1	0.584
chr07q	294001	296000	0	1.042
chr07q	323251	325250	0	0.894
chr07q	333001	335000	1	0.695
chr07q	335251	337250	0	1.067
chr07q	338001	340000	1	0.563
chr07q	346001	348000	0	1.308

chr07q	377001	379000	0	1.156
chr07q	391001	393000	1	0.753
chr07q	416501	418500	1	0.493
chr07q	449751	451750	0	0.958
chr07q	481251	483250	0	1.249
chr07q	500001	502000	1	0.580
chr07q	518501	520500	1	0.536
chr07q	536501	538500	1	0.472
chr07q	547501	549500	0	0.760
chr07q	547501	549500	1	0.760
chr07q	558751	560750	1	0.591
chr07q	562501	564500	0	0.712
chr07q	583501	585500	1	0.378
chr07q	592251	594250	0	0.944
chr07q	602751	604750	1	0.842
chr07q	639251	641250	0	1.113
chr07q	653001	655000	1	0.524
chr07q	666251	668250	1	0.311
chr07q	679751	681750	1	0.527
chr07q	682501	684500	0	0.923
chr07q	694001	696000	1	0.506
chr07q	711251	713250	0	0.899
chr07q	724501	726500	0	0.759
chr07q	747501	749500	0	NA
chr07q	823251	825250	1	0.490
chr07q	837751	839750	0	1.224
chr07q	860251	862250	0	0.853
chr07q	881251	883250	1	0.626
chr07q	914501	916500	1	0.718
chr07q	920751	922750	0	NA
chr07q	934001	936000	1	0.609
